# Effect of Bioprocessing on the Nutritional Composition, Antinutrients, Functional Properties, and Metabolites of Bambara Groundnut and Its Prospective Food Applications: A Review

**DOI:** 10.3390/molecules30112356

**Published:** 2025-05-28

**Authors:** Mpho Edward Mashau, Kgaogelo Edwin Ramatsetse, Thakhani Takalani, Oluwaseun Peter Bamidele, Shonisani Eugenia Ramashia

**Affiliations:** 1 Department of Food Science and Technology, Faculty of Science, Engineering and Agriculture, University of Venda, Thohoyandou 0950, South Africa; ramatke@unisa.ac.za (K.E.R.); thakhani.takalani@univen.ac.za (T.T.); oluwaseun.bamidele@univen.ac.za (O.P.B.); shonisani.ramashia@univen.ac.za (S.E.R.); 2 Department of Life and Consumer Sciences, College of Agriculture and Environmental Sciences, University of South Africa, Florida Campus, Johannesburg 1710, South Africa

**Keywords:** legume, nutritional quality, antinutritional factors, metabolites, processing methods, food applications

## Abstract

Bambara groundnut (*Vigna subterranea* (L.) Verdc.) is an underutilized leguminous crop, with its grains consumed differently, especially in developing countries. Bambara groundnut (BGN) is the cheapest source of protein and a rich source of dietary fiber, carbohydrates, amino acids, and minerals. It also contains a variety of non-nutritional components known as phytochemicals. The phytochemicals in BGN include polyphenols, flavonoids, tannins, phytic acid, oxalate, and trypsin inhibitors. Nevertheless, some phytochemicals are classified as antinutrients because they impair nutrient absorption. Bambara groundnut also contains metabolites, such as epicatechin, catechin, procyanidin, esters, and citric acid. Bioprocessing, such as dehulling, germination, malting, fermentation, ultrasonication, micronization, and others, reduces BGN flour’s antinutrients. However, bioprocessing may increase or decrease the levels of metabolites in BGN flour. For example, fermentation increases esters, whereas malting decreases them. Various studies have reported the use of BGN flour in bakery products, meat products, non-alcoholic beverages, pasta, and others. Thus, this study aimed to review the influence of bioprocessing on the nutritional quality, antinutrients, functional characteristics, and metabolites of BGN flour and its potential food applications. This study will explore the health benefits of bioprocessed BGN flour and promote its use in various food products.

## 1. Introduction

Bambara groundnut (*Vigna subterranea* (L.) Verdc.) is a legume species that originates from Africa and belongs to the *Fabaceae* family [1,2]. It is one of the cheapest sources of protein in developing countries such as Africa, Asia, and Latin America [3]. Bambara groundnut (BGN) is one of the less consumed legumes in the tropical part of Africa and is cultivated for its underground pods, producing respectable yields even under dry conditions and with low soil nitrogen levels [4]. The consumption of plant proteins is currently gaining popularity, and this is a result of the need to (partially) transition from diets high in animal protein to produce a more sustainable, wholesome, and reliable source of food [5,6]. Legumes, particularly pulses (dry seeds), are among the most significant plant groups that provide human protein intake after cereal grains and play a significant role in the traditional diets of many developing countries [7,8]. Other reasons that explain the importance of legumes in the diet include their protective effects against various chronic diseases, such as cardiovascular disease, type 2 diabetes, and cancer, and this is attributed to the consumption of legume antioxidants such as grain proteins [9]. Furthermore, the presence of phytochemicals has also encouraged the utilization of legumes because phytochemicals have been demonstrated to have positive health effects against diseases, such as cardiovascular and low-density lipoprotein levels [10].

**Figure 1:**
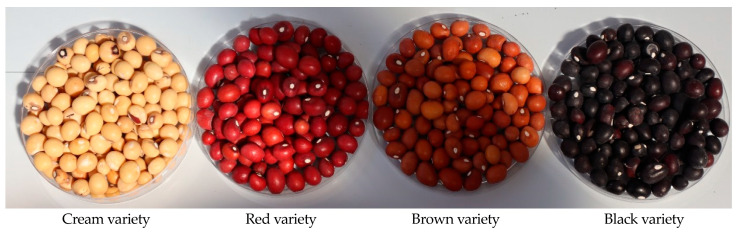
Different varieties of Bambara groundnut grains.

The grains of BGN have different colors, such as black, brown, red, and cream (Figure 1), and for a long time, fresh BGN were boiled or roasted and consumed as a snack [11]. Nonetheless, BGN is consumed differently depending on the country and tribe. In Botswana, immature grains are boiled with salt in their pods and consumed individually or mixed with boiled maize grains [12]. Furthermore, BGN is used to make cakes (‘*koki*’, food, paste, pounded) and eaten as a traditional bean (stew), where the grains are boiled, followed by frying with spices, salt, and flavors in oil in Cameroon [13].

Bambara groundnut is described as a complete food because it contains approximately 65% carbohydrates and a high protein content of 18% [14]. Moreover, BGN contains natural antioxidants such as flavonoids and phenolic acids and is more common in BGN with dark or red seed coats [15]. BGN also contains metabolites such as catechin, epicatechin, procyanidin, and citric acid [14]. Chemical characteristics such as the nutritional composition, bioactive compounds, and antioxidant activity of legume grains are influenced by bioprocessing, such as soaking, dehulling, malting, germination, and fermentation [16]. Oyeyinka et al. [17] found that the antioxidant activity of BGN was significantly reduced upon removal of the seed coat. Moreover, bioprocessing can also influence the modification of functional characteristics such as water and oil absorption capacities [18,19].

Bambara groundnut contains antinutritional factors, such as phytic acid, tannins, and trypsin inhibitors, which limit its incorporation into food products [20]. Antinutritional factors (ANFs) decrease the nutritional value of foods by binding to and lowering the bioavailability of minerals [14]. Phytic acid is available in large amounts in the seed coat and serves as a reserve of phosphorus for BGN. Condensed tannins are found in the testa and are more readily available in BGN with darker-colored grains. It reduces the bioavailability of minerals, proteins, and starch by forming indigestible complexes [21]. The retardation of protease activity can negatively affect the digestion of proteins and eventually hinder their absorption [22]. This review highlights the influence of bioprocessing on the nutritional composition, antinutrients, functional characteristics, and metabolites of BGN flours. It is anticipated that this review paper will raise awareness of processed BGN flour and its further applications, including its potential to be used as a functional ingredient in different food products.

## 2. Effects of Bioprocessing on the Nutritional Composition and Anti-Nutritional Factors of Bambara Groundnut

Bambara groundnut contains high amounts of protein and carbohydrates, as well as an appreciable amount of fat, ash, and fiber [8]. The protein content of BGN grains varies from 19% to 26%, while that of the flour ranges from 17% to 25%. Furthermore, the carbohydrate content of BGN grains ranges from 53% to 64%, while flour ranges from 32% to 62% [12]. The protein and carbohydrate contents of BGN are like those of other legumes, such as chickpea [23]. Goudoum et al. [24] determined the protein content of 11 varieties of BGN grains, and the cream had a higher value, whereas the red had a minimum value. Furthermore, Olaleye et al. [25] reported a higher protein content in dehulled BGN flour than in whole BGN flour. Table 1 shows the influence of bioprocessing on the nutritional composition of the BGN flour. The protein quality of BGN flour is higher than that of other legumes, and BGN flour contains high amounts of lysine and methionine with significant amounts of tryptophan, threonine, phenylalanine, isoleucine, and others [26]. The BGN flour contains a high crude fibre content, and the insoluble fibre content is higher than that of the soluble fibre. Nevertheless, BGN flour has higher quantities of soluble fiber than other legumes [27]. The black and red BGN varieties had high amounts of fibre, ranging from 23.9% to 24.3%, respectively, while brown eyes had a minimum value (17.7%). Bambara groundnut contains saturated fatty acids such as oleic and linoleic acids [28]. Palmitic acid is the third fatty acid in BGN, whereas linolenic acid is present in low amounts [29]. The BGN grains contain minerals such as iron, calcium, phosphorus, potassium, and sodium. Nonetheless, the bioavailability of minerals is affected by the ANFs available in BGN grains.

Oxalates, phytates, tannins, and protease inhibitors, such as BGN, are ANFs that are mostly present in legume grains. Antinutritional factors are harmful compounds that, when ingested in a product, disrupt the process of digestion and prevent the body from absorbing vital nutrients, particularly minerals, such as iron, calcium, and zinc. These minerals are primarily affected as ANFs bind to them to create complexes [30]. As a result, ANFS prevents grains from being utilized as efficiently as possible due to a decrease in the use of effective nutrients, which adversely affects their bioavailability [10,31]. Halimi et al. [32] indicated that ANFs in legumes could reduce their absorption and digestibility by approximately 50 percent. However, ANFs can be reduced by various processing techniques, such as soaking, fermentation, malting, and roasting.

**Table 1:** Effect of bioprocessing on the proximate composition of Bambara groundnut.

Bioprocessing	Moisture	Protein	Ash	Fat	Fibre	Carbohydrate	References
Roasting	Decreased (36.24)	Decreased (4.23)	Decreased (0.46)	Decreased (12.73)	Decreased (14.35)	Increased (6.65)	[27,33]
Soaking	Decreased (10.69)	Increased (10.61)	Increased (2.07)	Increased (12.69)	Decreased (22.31)	Decreased (1.85)	[34]
Fermentation	Increased (24.92)	Increased (9.46)	Decreased (17.95)	Decreased (63.57)	Increased (65)	-	[35]
Germination	Decreased (0.49)	Increased (10.31)	Increased (0.49)	Decreased (20.96)	Increased (13.09)	-	[36]
Malting	Decreased (23.31)	Increased (7.32)	Decreased (3.94)	Decreased (6.23)	Increased (43.54)	Increased (1.05)	[37]
Dehulling	Decreased (15.56)	Increased (15.76)	Increased (5.83)	Increased (38)	Increased (2.82)	Decreased (29.76)	[38,39]
Ultrasonicatin	Decreased (16.55)	Increased (12.49)	Decreased (1.21)	Decreased (21.59)	Increased (53.65)	Decreased (0.74)	[37]
Cooking	Decreased (54.02)	No change	No change	Increased (14.08)	-	Increased (7.14)	[38]
Boiling	Increased (120.33)	Decreased (5.68)	Decreased (11.80)	Decreased (27.11)	Decreased (21.20)	Decreased (7.55)	[33]

Values inside the brackets indicate the increase or decrease in the proximate composition of BGN as a percentage (%).

### 2.1. Roasting

Roasting is a traditional method for reducing antinutritive components and preparing grains for consumption. Based on the extent of the thermal treatment employed, roasting might change the amount of dietary fibre. It is a widely utilized processing technique that involves heating a food product, such as grain, to generate Maillard browning and caramelization for flavour [40]. Okafor et al. [41] reported an increase in the protein content of roasted BGN flour, which may have resulted from the enrichment of nutrients brought on by the reduction in moisture when roasted. The authors further found a decrease in moisture content with no significant variation in ash, fat, and fiber content. The decrease in the moisture content of roasted BGN flour may be due to the high temperature, which facilitates water evaporation from the grains, which can improve the quality of BGN flour [42]. Nonetheless, Ndidi et al. [33] observed a decrease in the fat and fiber contents of roasted BGN flour, while the carbohydrate content increased. The decrease in fat content may be related to the inhibition of lipase and lipoxygenase enzymes that initiate the oxidation of fatty acids [42]. Furthermore, the solubilization of polysaccharides during roasting may be associated with a decrease in fiber content [43]. The increase in carbohydrates in the BGN flour may be linked to the removal of unwanted compounds that may affect the carbohydrate content [44]. Adegunwa et al. [19] observed a decrease in calcium and magnesium and an increase in sodium, iron, and zinc contents in roasted BGN flour. However, Ijarotimi and Esho [45] reported a reduction in minerals of roasted BGN flour such as potassium, magnesium, phosphorus, sodium, and iron but an increase in calcium content. The improvement in some mineral contents may be due to the reduction in ANFs caused by roasting [46].

Bala and Rano [31] reported a significant reduction in the anti-nutrient content of roasted BGN flour, such as tannin, oxalates, and phytates (Table 2). The absorption and digestibility of calcium is reduced by phytates, which create calcium phytate complexes that prevent the absorption of iron. Phytates are reported to decrease the digestibility and absorption of minerals, hinder protein digestion by forming phytic protein complexes, and limit the absorption of nutrients by damaging the intestinal pyloric ceca [31]. The decrease in phytic acid content due to roasting may be related to the production of an insoluble complex between phytate and other compounds. Moreover, the decrease may be due to the inactivation of phytase activity or the formation of lower molecular weight at high temperatures (70–80 °C) [47]. The loss of compounds during roasting may be related to the reduction in tannins in BGN flour [48].

Furthermore, tannins may degrade or interact with other constituents of grain-like proteins to produce insoluble complexes, resulting in their decrease [54]. The reduction in oxalates in roasted BGN flour occurs because it is sensitive to heat and water solubility. Roasting increases the nutritional profile of BGN flours.

### 2.2. Soaking

The nutritional content of soaked grains can be affected both negatively and positively by the loss of nutritional composition and ANFs in water [7,55]. The coupling ability of biological molecules between cell matrices that may be adjusted by the temperature, acidity, neutrality, or alkalinity of water used to soak grains influences the speed and amount of lost ANFs and nutritional composition [29,55]. Mubaiwa et al. [56] found that soaking increased the protein content of red- and black-eye BGN cultivars, with no significant variation in moisture and fat content. The higher protein content of soaked red BGN flour than that of black-eye BGN flour indicates that the type of cultivar is affected differently by soaking. Furthermore, soaking may disintegrate complex compounds into simple and non-protein components, thereby increasing the protein content. Omoniyi et al. [34] reported a low moisture content in soaked BGN flours. The low moisture content of soaked BGN flour demonstrated that the flour may have longer keeping quality, since Usman [57] indicated that food samples with reduced moisture may have the advantage of extended shelf life. Furthermore, Omoniyi et al. [34] observed an increase in the fat content of soaked BGN flour, which was related to the leaching of soluble components that enhanced the amount of fat in the flour. Mazahib et al. [7] reported a decrease in iron, zinc, potassium, calcium, phosphorus, and sodium content of soaked BGN flour. A possible cause for the reduction in minerals such as calcium, iron, and potassium might be that they leached into the water used for soaking [58].

Researchers found a greater reduction in ANFs (Table 2) in BGN when soaked in heated liquid [19,54]. The loss of grain coverings (coats) and tannins (polyphenol substances that dissolve in liquid) from the cell onto the liquid accounts for the considerable loss of tannins in immersed products [54]. Similarly, Mazahib et al. [7] discovered extremely low concentrations of tannins in soaked BGN grains. This may be attributed to oxalate oxidase, which cleaves oxalic acid, leading to its leaching [59]. Tannins are phenolic substances that dissolve in liquids with high molecular masses and form difficult-to-digest compounds. Tannins limit protein availability by forming complexes and rendering them inaccessible. Soaking is a preparation step that requires little energy and is inexpensive but can considerably boost the utilization of BGN grains [29]. Moreover, soaking may also reduce ANFs, which impair the bioavailability of proteins. Omoniyi et al. [34] observed an increase in the fat content of soaked BGN flour, which was related to the leaching of soluble components that enhanced the amount of fat in the flour.

### 2.3. Boiling/Cooking

Bambara groundnut grains are usually cooked in a lot of water for a long time until they reach the required softness level. Diversity in gene frequencies, physical and chemical qualities, grain maturity, and storage temperatures may influence the time required to achieve the required finished products. Processing legumes by applying heat to water causes starch to swell and gelatinise, protein denaturation, and cell separation [60]. The nutritive value of boiled BGN grains differs based on the variety, prior treatments employed, and boiling duration. For instance, Ndidi et al. [33] discovered a decrease in the protein, ash, carbohydrate, and fat contents of boiled BGN grains. The grains were boiled for 3 h and 48 min in a seed-to-water ratio of 1:10 (*w*/*v*). The decreased protein content can be ascribed to the leaching of soluble proteins in cooking water [31]. Nonetheless, cooking did not increase the protein content of brown BGN grains [38] but slightly increased that of red BGN grains. An increase in moisture content may result in the inhibition of a significant amount of liquid by grains when boiled [61]. Oyeyinka et al. [38] observed an increase in the carbohydrate content of cooked BGN grains, whereas the ash content decreased. This increase in carbohydrate content may be associated with the release of oligosaccharides confined to other macromolecules during cooking. However, the decrease in ash content may be due to minerals leaching into the cooking water. The grains (250 g) were boiled in 2.5 L of water for 2 h and 30 min. Cooking generally decreases the mineral content of legume grains. The same authors observed a decrease in the potassium and phosphorus content of cooked BGN grains. Nonetheless, cooking did not affect the calcium, sodium, iron, and zinc content of the cooked BGN grains. Cooking enhanced most of the amino acid and protein digestibility of BGN grains. This increase in amino acids may be associated with the breakdown of some amino acids during cooking. Nonetheless, cooking decreased the methionine content of BGN grains. The reduction of ANFs during cooking, as well as the breakdown of the structure of long polymer chains of natural proteins, may be related to the increase in the protein content of BGN grains [62]. Adegunwa et al. [19] reported a decrease in calcium, magnesium, and zinc levels with an increase in the sodium content of boiled BGN grains. The decrease in mineral content may be due to the loss of minerals in boiling water. However, Ndidi et al. [33] did not observe any significant variations in iron, calcium, magnesium, sodium, potassium, and zinc levels in boiled BGN grains.

Boiling decreased the amount of ANFs, such as trypsin inhibitors, oxalate, hydrogen cyanide, and phytate (Table 2), which are beyond the standard range in raw BGN to acceptable standards. Research indicated that the nutritive content of boiled legumes is improved by inhibiting ANFs [33,54] and improving protein and starch in vitro digestibility [38]. The improved digestibility of protein is caused by inhibited ANFs, which are more vulnerable to moisture heating than to dry heating. This results in the liberation of the protein bound to ANFs, and the ability of starch digestion is enhanced by the breakdown of starch granules. Nonetheless, Pretorious et al. [14] noted a reduction in phytate and tannin content in BGN varieties cooked at 100 °C for 1 h and 30 min in a grain-to-water ratio of 1:10 (*w*/*v*). The cooked brown BGN grains had the highest phytate content of 1.6 mg/g while the cream had a lower value of 0.6 mg/g. The decrease in the phytate content of cooked BGN grains might be ascribed to phytate ions that might have leached into the drained and undetermined cooking water [14]. Furthermore, the cooked brown BGN grains had the highest tannin content (3.2 g/kg) while the orange BGN grains had the lowest value of 1.5 g/kg. The decrease in tannin content in cooked BGN grains might be due to the disintegration of the cell wall during cooking, which allowed the leakage of cell content [63]. Furthermore, phytate and tannins are vulnerable to heat and may be lost largely during soaking because they may leach with water [38].

### 2.4. Fermentation

Fermentation is traditionally a less advanced technique for processing legumes, which affects the nutritional content of BGN grains. Usually, entire grains are soaked, dehulled, cooked, and wrapped in banana leaves before fermentation for 4 days [29]. Adebiyi et al. [51] discovered that fermentation improves protein and essential amino acids in *dawadawa* made from BGN flour, with and without hulls. An improvement in protein content may be associated with the liberation of proteins and attachment to ANFs after fermentation, as well as with an improvement in the amino acids determined. Similarly, Igbabul et al. [64] argued that an enhancement in protein content might be due to an increase in microbial mass, which causes significant degradation of protein substances to amino acids and other peptides. The increase in protein content of *dawadawa* samples can also be due to fermenting microorganisms having greater accessibility to the interior parts of the BGN, which contain a considerable amount of protein [65]. Arise et al. [66] also found a greater protein content in fermented BGN flour than in soaked and boiled BGN flour. The enhancement of protein in fermented BGN flour may be related to the enzymatic hydrolysis of peptides into free amino acids. Sobowale et al. [35] observed an increase in the moisture, protein, and crude fiber content of fermented BGN flour, whereas ash and fat contents were reduced. The authors indicated that the increase in moisture content may be associated with the addition of water to the substrate prior to fermentation. Furthermore, the increase in fiber content may be linked to the enhanced utilization of other components, such as starch, during fermentation [37]. Chude et al. [67] reported a rise in fiber content of fermented BGN flour, possibly because of the activities and degradation of microorganisms used for fermentation, as well as partial dissolution of cellulose and hemicellulosic substances by microbiological enzymes. As a result, lower crude fiber is recommended because indigestible fiber may attach to minerals and act as a barrier for enzymes used for digestion, reducing the absorption and digestion of vital minerals and jeopardizing nutritional security [68]. The decrease in fat content of fermented BGN flour may be due to the enhanced activity of lipolytic enzymes, which disintegrate fat into fatty acids and glycerols [69]. The reduced fat content is desirable because fermented BGN flour has a longer storage life due to minimal lipid oxidation. The reduced ash content of BGN flour may be ascribed to the release of soluble inorganic salts during fermentation [70].

Fermentation increases the mineral content of BGN flour, including potassium, phosphorus, magnesium, and zinc, as reported by Sobowale et al. [35]. The increase in minerals is related to fermentation, which increases their bioavailability. This is because milling BGN grains into flour before fermentation improves the surface area of the grains and interrupts the cellular structure, releasing phytase, which disintegrates phytate [71]. Onuora and Iro [72] reported a reduction in the calcium, zinc, phosphorus, sodium, iron, and copper content of BGN flour fermented with *Lactobacillus plantarum* and *Lactobacillus fermentum*. The authors indicated that calcium might have leached into the drained water, which has been utilized for fermenting the sample, leading to its reduction. They further suggested that the decrease in iron content may result from the release of iron into the fermentation medium. However, Ijarotimi and Esho [45] reported an increase in the calcium and iron content of fermented BGN flour. Similarly, Ijarotimi et al. [73] reported an increase in the calcium and iron content of naturally fermented BGN flour. Therefore, from the results obtained, naturally fermented legumes are more recommended than inoculated fermentation in terms of improving the mineral content of legumes.

Fermentation has been reported to reduce ANFs, as shown in Table 2. Chude et al. [67] reported a significant decrease in the phytic acid content of fermented BGN flours. The dissolution of ions in the fermenting liquid beneath the variation in the chemical capability that controls the pace of diffusion can be the cause of the inhibition of phytates in fermenting BGN flour. Furthermore, the reduced phytic acid concentrations may be due to the enzymatic reactions of fermenting microbes that degrade phytic acid or the complexes they produce [74]. However, other preparation procedures, such as soaking and boiling before fermentation, may also have contributed to the decrease in the phytic acid content. Olukomaiya et al. [75] indicated that fermentation enhances the activity of phytase enzymes, which can degrade non-soluble organic complexes with minerals, thereby decreasing phytic acid. Furthermore, microorganisms produce phosphatase enzymes that can break down phytic acid [76]. Phytic acid is linked to difficult-to-cook phenomena in legumes and the binding of other important nutrients [77]; therefore, it is important to eliminate it in legumes. Oxalate is difficult to dissolve at gastrointestinal pH, whereas tannins bind strongly with enzymes that aid in digestion and some micronutrients, thereby lowering their digestibility [78]. Nevertheless, when fermenting legumes, ANFs may be broken down, thereby decreasing their content and enhancing nutritional digestibility [79]. For example, the activities of microorganisms during fermentation may account for the reduced oxalate content of fermented legume flours, such as BGN flour. Nonetheless, Emammbux and Taylor [80] reported that the activity of the phenyl oxidase enzyme during fermentation reduces the tannin content. Sobowale et al. [35] noted a decrease in the trypsin inhibitor content of fermented BGN flour, which was associated with the disintegration of trypsin inhibitors during fermentation.

### 2.5. Germination and Malting

Germination is an expensive and efficient method that reduces cooking challenges, including shortening cooking times, improving nutritional value, and removing or lowering ANFs [81]. In terms of nutrition, Chinma et al. [36] reported an increment in the protein and fiber content of germinated BGN flour. According to Xu et al. [82], the improvement in protein content might be due to the production of enzymes by the germinated grains, compositional shift brought on by the breakdown of other components, and synthesis of novel proteins. Moreover, the disintegration of protein–antinutrient complexes or degraded components other than proteins during germination may enhance the protein content [83]. In contrast, Okafor et al. [50] reported no significant differences in protein, moisture, ash, fat, or fiber content. Chinma et al. [36] observed an increased fiber content in germinated BGN flour, which was related to the production of new principal cell walls and resistant starch. Therefore, the disparity between the results might be due to the type of cultivar or the analysis procedure used. In addition, the variation in fiber content may be due to the difference in thickness of the grain coat, since fiber is mostly found in the grain coat [84].

Nonetheless, Mudau and Adebo [37] observed a high fiber content of the malted BGN grains, which was related to the enhanced utilization of other components such as starch during germination. The same authors reported a reduction in the fat, ash, and carbohydrate contents of malted BGN flour. The reduced ash content of malted BGN flour may be related to the leaching of water-soluble minerals during the soaking of BGN grains [85]. Disintegration of starch granules by hydrolytic enzyme activity may be associated with the reduction in carbohydrates during malting. Moreover, this decrease may be due to the degradation of carbohydrates into simple sugars during malting, which is promoted by the activity of natural enzymes such as α-amylase, which improves digestibility [86]. Germination increases some of the minerals found in BGN flours, while it also decreases others. For example, Chinma et al. [36] reported an increase in calcium, potassium, phosphorus, and magnesium levels in germinated BGN flours. However, the iron, sulfur, and phosphorus levels decreased. The enhanced mineral content of BGN flour may be linked to the liberation of bound minerals and the reduction of ANFs during germination [87]. Furthermore, the essential and non-essential amino acids in the germinated BGN flour were higher than those in the control sample. The increase in amino acids is linked to germination and reduced ANFs. The protein digestibility of germinated BGN flour increased, which was linked to germination, activating the digestion of grain proteins, thereby increasing the digestibility of protein.

Germination decreased the ANFs of BGN flour (Table 2). The decrease in phytate content may be due to phosphatases that break down phytate and produce substances such as inositol during germination [88]. Furthermore, the hydrolytic activity of the phytase enzyme may also contribute to the reduction in phytate of germinated BGN flour because when the activity of phytase increases, the content of phytic acid decreases [41]. Phytates decrease the chances of minerals such as calcium, iron, and zinc being absorbed by the human body [36]. Besides the negative effects of phytates, Adebo et al. [89] indicated that they possess health-promoting properties, such as anticancer and antioxidant properties. Nonetheless, the decrease in tannin content in germinated flour is associated with the leaching of soluble tannin components during soaking and the degradation of tannin by natural enzymes. The degradation of proteins by enzymes such as protease inhibitors, which inactivate proteinaceous antinutritional compounds such as trypsin inhibitors, may be attributed to the decrease in trypsin inhibitors in germinated BGN flour [36].

### 2.6. Dehulling

The removal of the grain outer covering, known as dehulling, has been reported to enhance the taste, palatability, and reduce the ANFs of legume grains [90,91]. Dehulling can also affect the nutritional composition of BGN grains. Nti [92] and Abiodun and Adepeju [18] reported an increase in the protein content of dehulled BGN grains. Consequently, an improvement in the constituents of the BGN interior layers after the removal of hulls might be the cause of the increase in protein levels [17]. Furthermore, Elochukwu (2020) [93] reported a reduction in fiber and ash content of dehulled BGN grains, possibly due to the elimination of some of their bran and grain coat, which might contain some of the minerals and fiber content. Yahaya et al. [39] reported a decrease in the moisture content of dehulled BGN flour and indicated that removal of the seed coat makes it difficult for water to penetrate; hence, its removal allows for imbibition. The increase in the fat content of dehulled BGN flour is related to the removal of the portion of the hull and the concentration of the endosperm.

Dehulling has been reported to increase and decrease the minerals of BGN flour. Abiodun and Adepeju [18] reported a reduction in calcium, magnesium, potassium, phosphorus, sodium, iron, and zinc levels in dehulled BGN flour. The decrease in the mineral content may be due to the removal of the seed coat of the grains [77]. However, Nti [92] reported no consistent trend for different dehulled BGN cultivars. For example, the calcium content of the black white-eye BGN variety increased, and that of the cream pink-eye variety decreased, with no significant difference in brown white-eye and maroon white-eye varieties when compared to their specific control varieties. In addition, the phosphorus content increased in the cream black eye, cream pink eye, brown–white eye, maroon–white eye, and decreased in the black–white eye varieties. Similarly, Yahaya et al. [39] reported an increase and decrease in the sodium content of dehulled cream and red BGN flour, respectively. In addition, there was a decrease and an increase in the potassium and iron content of cream and red BGN flour, respectively. However, the calcium content decreased in both varieties. Differences in the mineral content of different BGN varieties may be caused by several factors, such as environmental factors (soil, rainfall, and seasons), processing aspects (temperature, storage duration, and preserving technique), and plant features (age, variety, maturity, and species) [94].

Dehulling has been reported to reduce the ANFs, as shown in Table 2. Given that the majority of ANFs are in the outer coating of the grain, their decrease may be due to the removal of the grain coat [39]. Furthermore, according to Oyeyinka et al. [17], hulled BGN grains contain more phytates than dehulled (cotyledons) BGN grains, which could account for the greater decrease in phytate concentrations of dehulled grains. Similarly, Yahaya et al. [39] reported a significant reduction in the phytate content of dehulled BGN flours. This reduction was associated with the dehulling of the grains because most of the ANFs are available on the hull or seed coat. Oyeyinka et al. (2019) [38] reported a decrease in the tannin content of dehulled BGN flours. Tannins are present in high amounts in the seed coat and impair the bioavailability of proteins and minerals.

## 3. Effects of Processing Methods on the Functional Properties of Bambara Groundnut Flour

### 3.1. Roasting

Roasting of protein-rich foods, such as legumes, leads to alterations in the moisture content, solubility, and functional properties of proteins (Collar et al., 2020) [95]. Table 3 shows the effect of roasting on the functional properties of BGN flour. Roasting increases the water absorption capacity (WAC), and there is no change in the oil absorption capacity (OAC), forming capacity, emulsion capacity (EC), and bulk density (BD). The increase in WAC is most likely a result of protein denaturation caused by roasting, exposing hidden hydrophilic sites and enabling them to interface with water [91]. Legume flour with an excellent ability to absorb oil can be used as a flavor preserver in a variety of food products, and legume flour with a greater ability to absorb water can be used for product development, particularly for gravies, baked goods, soups, and doughs [96]. The decrease in the forming capacity of roasted BGN flour is the result of protein denaturation caused by protein network formation at extremely high temperatures, thereby reducing the solubility and adaptability of the protein [42]. The reduction in EC is likely due to potential protein changes that occur after exposure to heat [97]. The reduction in BD after roasting might be due to the formation of a permeable structure, which increases the porosity and subsequently decreases the moisture content owing to the heat treatment [98]. The low BD of BGN flour is beneficial for producing complementary newborn and weaning food [61].

### 3.2. Soaking

Table 3 shows the effect of soaking on the functional properties of the BGN flour. Soaking increases WAC and OAC but decreases the forming capacity, emulsion capacity, and BD. The ability of BGN flour to absorb more water may be due to an alteration in the protein makeup and loosening of the starch polymer caused by soaking of the grains [91]. In addition, the increase in WAC and OAC may be attributed to an increase in the amount of overall protein, which is both water-friendly and hydrophobic and can react with water and oil in a product [99]. Mazi et al. [100] reported that prolonged soaking times resulted in increased moisture content in the grains, which could cause more gelatinization when dried at higher temperatures, ultimately leading to greater WAC. The water absorption capacity refers to the capacity of flour to absorb water and create an enhanced texture culinary product. It is also beneficial for improving product output, textural properties, and structure in food chains, and improves product functionality in emulsion processes [101]. Furthermore, the ability of flour components to physically bind oil through complicated capillary binding processes is essential for a variety of processes, which improves flavor, mouthfeel, and consistency [61].

Soaking improves foaming capacity because it establishes a condition that allows starch and protein components to absorb a greater amount of water, which promotes the development of foam [102]. Foaming capacity is a desired functional property in food systems that enables its application in whipped toppings, frozen dessert mixtures, and baked products. Foaming capacity develops when proteins are beaten up, producing a surface layer that keeps the air bubbles suspended and decreases the mixture [103]. Bulk density is an important property that aids in selecting the proper packaging material [104]. The reduction in BD occurs because more soft molecules are produced when grains are soaked as they increase their moisture content and loosen their structure [96]. Because soaked BGN flour has a reduced BD, it can be utilized in complementary foods because it lacks a thick texture and greater viscosity [105]. However, legume flour with greater BD is useful for creating foods that are easily dispersed, such as baby foods [106]. Furthermore, since a greater BD of flour may improve lipid absorption, it may be utilized in baked and pastry foods [53].

### 3.3. Boiling

Table 3 depicts the influence of boiling on the functional characteristics of the BGN flour from raw to boiled. Boiling increases the WAC and decreases the forming capacity, emulsion capacity, and BD. The enhanced WAC of BGN flour may be due to expansion of the starch–protein complex structure [107]. The application of heat can result in protein unfolding by weakening the noncovalent linkages in the secondary and tertiary structures of proteins, thereby improving WAC [61,108]. Arise et al. [108] reported an increase in OAC of boiled BGN protein isolates from 56 to 95.50%. The physical structural variations in boiled legume flour can result in increased porosity, which enables more lipids to be trapped. The notable reduction in FC may be the result of high temperatures, which result in the unfolding of proteins [109]. In addition, Gandhi et al. [102] stated that the decrease in FC of legume flour might be because various constituents of the food matrix react upon boiling, which lowers the capacity of the product to generate foam.

### 3.4. Fermentation

Table 3 shows the effect of fermentation on the functional properties of raw and fermented BGN flours. Fermentation increases the WAC and foaming stability and decreases the OAC, foaming capacity, and emulsion capacity. The increase in the WAC of BGN flour may be due to the unfolding of protein and starch breakdown throughout the fermentation process [35,110]. The reduction in OAC of fermented BGN flour suggests that throughout the fermentation process, the conformational modification of starch and protein molecules resulted in fewer hydrophilic and hydrophobic sites [35]. The capacity of lipids to bind is largely dependent on the presence of nonpolar amino acids. Throughout the fermentation process, the arrangement of protein molecules is altered, changing the surface available for these amino acids and causing either an improvement or reduction in OAC [111,112]. The decrease in EC may be due to the inability of the protein to bind to fat throughout the fermentation process, which may be problematic in certain processes where foaming is preferred [35]. In addition, according to previous research, *Lactobacillus* may cause alterations in the hydrophobic protein sites, which may result in a reduction in emulsifying qualities because microorganisms generate alcohol during fermentation [113,114]. This inhibits migration to the surface and prevents the production of foams and emulsions [115]. The decrease in the foaming capacity of BGN flour may be linked to the hydrolysis of protein during fermentation because it maintains the suspension of air bubbles [116]. Furthermore, the decrease in EC of fermented BGN flour may be due to the inability of the protein to interact with oil, which can be a problem where aeration is advantageous [117].

### 3.5. Germination and Malting

Table 3 shows the influence of germination/malting on the functional characteristics of BGN flour. The germination process enhances the functional qualities of grains, which influences the preparation, uses, characteristics, acceptability, and production of possible ready-to-consume snack meals [118]. It is a low-cost technique to boost cereal grains and pulses’ nutritional and functional properties. Grains naturally germinate when they sprout, causing starch to be broken down by enzymes such as amylase to produce dextrin and maltose [119]. This technique modifies structural components, making it simpler to create new bioactive compounds and greatly enhancing legume nutritional content, accessibility, and resilience [120,121]. Germination increases WAC, OAC, BD, and forming capacity and decreases forming stability and OAC. The improvement in WAC can be the result of macromolecule modifications, which result in more hydrophilic parts. Moreover, throughout the germination process, starch polymer structures are lost and transformed into monomers, which enhances the capacity of the germinated BGN flour to absorb water [34,36]. The increased WAC of the germinated BGN flour may be linked to an increase in protein content and alterations in protein quality during germination. Thus, the enhanced WAC of BGN flour will allow more water to be added to the dough during baking, which will improve handling and maintain the freshness of baked products. In contrast, Mudau and Adebo [37] observed a decrease in the WAC of malted BGN flours.

The decrease in the OAC of BGN flour throughout germination may result from water-loving sites being lowered by the enzymatic breakdown of fiber and starch [118]. Furthermore, the reduction in OAC of germinated BGN flour may be associated with the effect of germination on protein hydrophobicity [122]. Chinma et al. [36] further indicated that the higher OAC of unprocessed BGN flour may be due to its hydrophobic protein and lipid composition, which are implicated in fat absorption. Nonetheless, Mudau and Adebo [37] did not observe a significant variation between the OAC of raw and malted BGN flours. The rise in the forming capacity of BGN flour may be related to the hydrolysis of protein molecules throughout the germination process to generate a small molecular weight subunit structure, thereby increasing protein solubility and strengthening the absorption ability of the air–water interface [123]. The changes in the structure of macromolecules, including dietary fiber, protein, and starch throughout germination, may contribute to the reduction in the BD of BGN flour [124]. This decrease in the BD of germinated BGN flour may be useful when preparing low-bulk-weaning food. Similarly, Mudau and Adebo [37] observed a decrease in the BD of malted BGN flour and indicated that the degradation of proteins and carbohydrates to simpler forms during malting contributed to the decrease in BD.

### 3.6. Micronization

Micronization is an alternative approach used to quickly heat a product that uses electromagnetism and infrared heat in the near-infrared region. Owing to the rapid generation of elevated temperatures, this approach alters the structural makeup of biomolecules in different ways, which affects their functional characteristics [125]. Ogundele et al. [126] reported a significant decrease in the swelling index and swelling power of micronized whole and dehulled BGN flour with an increase in micronization time and temperature compared to whole BGN flour. The authors further indicated that the degree of swelling was greater in dehulled BGN flour than in whole BGN flour, which may be due to variations in their chemical makeup, such as dietary fiber. These results are also in line with those of Mukwevho and Emmambux [127], who reported a reduced water-swelling index and increased WAC of micronized BGN flour. Studies on the impact of micronization on the various functional characteristics of BGN flour are limited. However, according to previous studies, micronization improves the functional properties of some crop products, such as wheat bran [128], black gram by-product [125], green pea, yellow lentil, kabuli chickpea, and red lentil powder [129]. Furthermore, micronization has negative effects on the functional properties of barley protein concentrates [130,131], navy bean, [131] and chickpea flour [132].

### 3.7. Ultrasonication

Ultrasonication is a novel and environmentally friendly technique that modifies proteins [133]. Ultrasonication is a suitable non-thermal approach for improving production and leads to minimal adverse effects on the physicochemical, structural, and functional characteristics of food products [134,135]. This approach uses sound waves with frequencies greater than 16 kHz. It is classified into two categories: low frequency, which ranges from 16 to 100 kHz, and high frequency, which ranges from 100 kHz to 1 MHz [136]. Throughout this procedure, proteins undergo significant modifications in their molecular makeup and functional properties because of the strong disruptive pressures that break them down into tiny molecules [137]. Mudau and Adebo [37] reported an increase in the WAC, OAC, and swelling capacity and a decrease in the BD of ultrasonicated BGN flour. The improvement in WAC and OAC may be because ultrasound exposes proteins to the external environment, especially their hydrophobic and hydrophilic sites, which improves their capacity to retain fat and water [138]. The enhanced WAC of ultrasonicated BGN flour may be related to the hydrophobic regions and the thiol group of the amino acid being exposed to ultrasonication, leading to an enhanced capacity to absorb water [139]. On the other hand, the enhanced OAC may be linked to the hydrophobic regions of the protein being exposed to the outside condition following ultrasonication, thereby enhancing their capacity to physically trap oil [138]. The decreased BD of ultrasonicated BGN flour may be associated with the degradation of proteins and carbohydrates into simpler forms during ultrasonication [37]. The reduced BD of ultrasonicated BGN flour demonstrates its potential for use in the formulation of low-BD weaning foods for infants. The increase in the swelling capacity of ultrasonicated BGN flour showed a greater affinity for water, and the flour produced food products of high quality.

## 4. Effects of Processing Methods on the Metabolites of Bambara Groundnut

Bioactive compounds are secondary metabolites obtained from natural sources, such as grains, fermented products, and foods that contain greater amounts of lipids or have the potential to promote the well-being of humans [140,141]. Since the medicinal qualities of many physiologically bioactive components found in different BGN varieties have been extensively studied, they are portrayed as a source of products that promote well-being and may be utilized as a significant alternative nutritious food [27,142]. Several researchers have demonstrated that these compounds are found in BGN grains such as catechin dimer, catechin glucoside, medioresinol, epicatechin, salicylic acid, p-coumaric acid, ferulic acid, caffeic acid derivatives, quercetin-3-O-glucoside, quercetin, 3-O-rutinoside, and myricetin hexoside [27,143]. Harris et al. [144] found the most significant compounds, such as quercetin, kaempferol, rutin, myricetin, methyl gallate, catechin, chlorogenic acid, ellagic acid, and gallic acid, in BGN flour. Different analytical equipment is used to measure the metabolites of bioprocessed BGN flour, including gas chromatography/high-resolution time-of-flight mass spectrometry (GC–HRTOF-MS), ultra-performance liquid chromatography–quadrupole time-of-flight mass spectrometry (UPLC–QTOF-MS), and gas chromatography–mass spectrometry (GC–MS).

The metabolites in BGN flour can be affected by different processing methods, such as cooking, dehulling, malting, ultrasonication, infrared, and roasting, as indicated in Table 4. Oladimeji and Adebo [11] reported that when BGN grains are processed, novel metabolites that are not present in the raw flour may be produced or detected. Nonetheless, according to the results obtained by Mudau et al. [145], some metabolites present in raw BGN can be increased, destroyed, or decreased depending on the duration of processing. For example, Oyedeji et al. [146] detected butylated Hydroxytoluene, Squalene, Stigmasterol, and Glycerol on days 0, 1, 2, and 3 of germination. Furthermore, Oyedeji et al. [146] reported that metabolites obtained through the germination of BGN flour revealed that substances found in them may provide a deeper comprehension of their components and may be utilized to produce functional foods. Nyau et al. [143] profiled the phenolic compounds in germinated BGN and found that the overall content increases by 1.3 times with germination, indicating the emergence of novel metabolites.

## 5. Selected Methods Used to Measure the Bioactive Compounds of Bambara Groundnut

Different analytical methods have been used to measure the polyphenol content and antioxidant activity of BGN flour. The Folin–Ciocalteu method is a standard method used to measure the total phenolic content (TPC) and total flavonoid content (TFC) of BGN flour. Methanol is mostly used to obtain BGN flour extracts for the measurement of the TPC, TFC, and antioxidant activity of bioprocessed BGN flour. To measure the antioxidant activity of bioprocessed BGN flour, various methods, such as ferric reducing antioxidant power (FRAP), oxygen radical absorbance capacity assay, ABTS, and DPPH radical scavenging assays, are used. Mudau and Adebo used methanol extract and observed an increase in the TPC of fermented BGN flour, while malting and ultrasonication decreased the TPC of the flour. The increase in TPC of fermented BGN flour is possibly related to the phenolase enzyme, which may have released bound phenolics [158]. The decrease in the TPC of malted BGN flour is likely associated with the phenolase enzyme, which may have hydrolyzed the phenol components during steeping and malting. In contrast, Chinma et al. [36] reported an increase in the TPC of germinated BGN flour, which may be related to the activation of enzymes that break down the cell wall and thereby alter its structure. This phenomenon leads to the liberation and biosynthesis of polyphenols during malting [159]. In contrast, bioprocessing (malting, fermentation, and ultrasonication) enhanced the TFC of BGN flour. The increase in the TFC of bioprocessed BGN flour is likely due to the production of enzymes during steeping prior to malting, the activities of microbial enzymes during fermentation, and the cavitation phenomenon during ultrasonication [37]. Regarding antioxidant activity, fermentation increased the ABTS and FRAP assays of BGN flour, whereas malting and ultrasonication decreased antioxidant activity. The increase in antioxidant activities of fermented BGN flour may be associated with the production of enzymes due to the activities of microorganisms, fermenting microorganisms, metabolizing polyphenols, and the liberation of earlier bound polyphenols [160]. Adedayo et al. [161] used methanol extracts to determine the TPC, flavanol, flavonol, flavanol, anthocyanin, and antioxidant activity of whole and dehulled BGN flours. Dehulling decreased the TPC, flavonol, flavanol, and anthocyanin content of BGN flour. The reduced TPC of the dehulled BGN flour may be related to the removal of the outer layer because it is known to contain high amounts of polyphenols [162]. A similar trend of decrease in flavanol and flavonol content of the dehulled BGN flour was observed. Furthermore, dehulling reduced the FRAP and oxygen radical absorbance capacity of the BGN flour, which may be related to the removal of the outer layer of the BGN grains. Oyeyinka et al. [38] also used methanol extracts to measure the TPC and antioxidant activity of cooked BGN grains. Cooking increases the TPC of BGN grains, and this is related to conjugate dissociation due to cooking, which leads to an increase in the total fraction of polyphenols [163]. Furthermore, cooking decreased the DPPH and ABTS activities of the BGN grains. The decrease in antioxidant activity was associated with the leaching of soluble antioxidants during steeping prior to cooking. The seed coat of BGN also influences the degree and rate of leaching of polyphenolic compounds [164].

## 6. Effect of Bambara Groundnut on the Physicochemical and Sensory Attributes of Food Products

Bambara groundnut has pleasant sensory attributes, particularly in terms of flavour [165]. Therefore, BGN flour has been incorporated into food products to enhance their nutritional and sensory qualities (Table 5). Nevertheless, it is important to keep in mind that not all customers can explore new products. Other customers are hesitant to explore products that contain unique components or are made using new technologies [166,167]. Product neophobia consumers’ segmentation enables the development of strategies based on the preferences of individuals who are more prepared to test novel or modified products [168]. It is broadly understood that new food formulations should focus on consumers’ interest in healthy food. Sensory analysis of novel products is critical to their success. Researchers have examined individuals’ perceptions of new products, and most researchers have focused solely on product features [169]. Improving the nutritional composition of products while preserving consumer approval is thus achievable. For example, Denhere [165] found that brown and red BGN flours improved the overall acceptability of instant provitamin A-bio-fortified maize. However, red BGN flour decreased the average score of colour and texture, while brown BGN flour improved the average score of all the sensory attributes of the samples as compared to the control. Ayo and Andrew [170] found that adding BGN flour (10%) improved the overall acceptability of acha-date palm biscuits but decreased at levels of 15% to 25%. However, other sensory attributes were highly favoured by the inclusion of BGN, except for colour.

Bambara groundnut flour has been incorporated into biscuits [171], infant formula [172], and steamed bread [173]. However, BGN has been reported to contain little oil, and it is suggested to be used for human consumption and incorporated in animal feed because of its high carbohydrate and protein content. The supplementation of millet biscuits with 15% and 30% BGN flour improved the protein content and color [174]. Adding 10% BGN flour did not affect the wheat bread quality [175]. Similarly, Olapade et al. [176] found that including 10% BGN flour in *fufu* was highly favoured based on sensory ratings compared to control and higher inclusion (20–50%) of BGN flour. For overall acceptability, Atoyebia et al. [177] discovered that people preferred BGN products such as BGN paste (*okpa*), BGN beans, and BGN milk only, rather than some snacks. Anaemene et al. [51] reported an improvement in the nutritional composition and a decrease in the sensory properties of muffins with increasing substitution levels of germinated BGN flour. This suggests that adding BGN flour to snacks increases their nutritional content but undesirably affects their sensory attributes due to its stronger beany flavor. Bravo-Núñez and Gómez [178] suggested that an excessive level of legume flour should not be utilized when making snacks because of their bitterness and bad flavor.

**Table 5:** Application of Bambara groundnut flour in selected food products.

Products Produced	BGN Incorporated (%)	Effects of BGN on Overall Product Quality	Reference
Fermented maize flour	30	Increased protein, fat, fibre, and ash, as well as decreased moisture and CHO content Reduced anti-nutritional level (tannins and trypsin inhibitors)	[170]
Acha-date palm-based biscuit	2.19 to 10.94	Increased protein, fat, ash, and fibre, as well as moisture and CHO, with an increase in their level.	[179]
Cakes prepared from wheat and raw plantain powder	2.49 to 12.47	Increased fat, ash, fibre, and energy, as well as decreasing moisture, CHO	[180]
Maize flour	10 to 20	Increased protein, fat, and fibre content Increased functional properties (bulk density, swelling capacity, water holding and binding capacity)	[181]
Traditional maize-based snacks: Ipekere Agbado	10 to 30	Increased protein, fat, ash, and fibre content	[182]
Maize-based pudding	4.81 to 40	Increased moisture, ash, and protein, as well as decreased CHO and fat content. Increased bulk density, swelling index, and water absorption capacity. Mesophillic bacteria count increased	[183]
Maize Tortillas	5 to 20	Improved total phenolic content, total flavonoid content, ferric reducing antioxidant power, and 2,2-diphenyl-1-picrylhydrazyl (DPPH) Increased moisture, ash, protein, fat, and fibre content, as well as decreased CHO content.	[184]
Maize snack (kokoro)	0.2 to 30	Increased protein, moisture, ash, and fat, as well as decreased fat, fibre, and CHO content. Increase bulk density, as well as decrease water absorption capacity, oil absorption capacity, and swelling capacity with an increase in its level.	[66]
Provitamin A *mahewu*	30	Roasted and germinated flour increased protein, fat, and ash, as well as decreasing CHO and moisture content. All flours increased the amino acid profile.	[185]
Rice crackers	9 to 18	Increased protein, CHO, fat, as well as decreased fibre, moisture, and ash content. Decreased water absorption index, bulk density, oil absorption capacity, forming stability and did not affect forming capacity and true density with increase in its level.	[186]
Wheat noodles	20	Increased protein, fat, fibre, ash, and mineral content (Magnesium, phosphorus, potassium, sodium, and sulphur), as well as decreasing CHO content.	[187]
Sorghum *mahewu*	20 to 30	Increased protein, fat, and fibre, as well as a decrease in ash content, with an increase in its level. Initially decreased CHO content with its increase (20%), but further decreased with its increase (30%)	[188]
Wheat bread	0 to 40	Increased protein, fat, ash, fibre, and moisture, as well as decreased moisture content. Increased mineral content (sodium, potassium, calcium, phosphorus, and iron) Increased water absorption capacity, foaming capacity and stability, as well as decreasing oil absorption capacity and swelling index	[189]
Mutton patties	2.5 to 10	Enhanced fibre and ash contents, but moisture, protein contents, and carbohydrates were reduced. Delayed lipid oxidation was observed when compared to the control sample. Improved the technological and textural properties such as cooking yield, hardness, and resilience	[190]
Wheat rusk	5 to 20	Enhanced ash, protein, fat, and crude fibre content, physical characteristics such as hardness, loaf volume and specific volume, and fracturability decreased	[191]

BGN: Bambara groundnut, CHO: Carbohydrates.

Bambara groundnut has also been used in meat products, and Alakali et al. [192] found no significant difference between the sensory properties of non-formulated and BGN flour-formulated beef burgers over a 21-day storage period. Argel et al. [193] found that employing legume flour as a partial pork substitute improved the overall acceptability of patties. Ramatsetse et al. [190] added 10% of three varieties of BGN (brown, cream, and red) flour to mutton patties, and the sensory score of formulated patties, such as aroma, texture, appearance, and overall acceptability, did not differ from the control sample. Nevertheless, mutton patties added with 10% brown BGN flour and cream BGN flour had lower sensory scores compared to the control sample. Panelists liked the mutton patties added with 10% red BGN flour, as it was like a control sample in most of the sensory attributes. Therefore, including 10% BGN flour in other food products is recommended.

On the other hand, Akanni et al. [194] added BGN flour at levels of 20 and 30% to improve the metabolites and storage stability of sorghum *mahewu*. Panelists preferred *mahewu* added with 20% BGN flour, but this was lower than the control sample. The low sensory parameter scores were related to the availability of metabolites, such as hexadecanoic acid methyl ester and octanoic acid 2-dimethylaminoethyl ester. The taste of the former is oily and waxy, while the latter has an odor that is musty and pungent. The dominant metabolites in blended *mahewu* samples were esters because they are produced when fatty acids and alcohols chemically interact and when the metabolites of alcohol interact with organic acids [195]. The blended *mahewu* samples had high amounts of organic acids and low amounts of alcohol compared to the control sample. Organic acids are important in fermented products for the development of flavors [196]. The low alcohol content of the blended *mahewu* samples may be related to lactic acid fermentation, which can decrease the amount of alcohol while producing different esters [195]. *Mahewu* supplemented with 30% BGN flour had higher amounts of alkanes, alkenes, and benzene derivatives, whereas *mahewu* supplemented with 20% BGN flour had greater amounts of aldehydes and ketones. In terms of storage stability, there was no mold or yeast growth on the blended *mahewu* samples during the storage period of four weeks.

Defatted BGN flour and milk have been added to food products. Arise et al. [196] substituted wheat flour with up to 30% BGN protein isolate for noodle production. The proximate composition of the composite noodles, including protein, moisture, and ash, increased. Furthermore, amino acids, cooking yield, and cooking loss of noodles increased with increasing substitution levels of the BGN protein isolate. Sensory evaluation of noodles demonstrated that up to 25% BGN protein isolate could be added to noodles because this formulation had higher scores for aroma, color, texture, and overall acceptability. Bambara groundnut milk has been used as a substitute for cow’s milk in dairy products. Balogun et al. [197] added 10–40% BGN milk to yoghurt and protein, and ash content increased with the substitution levels but decreased after four weeks of storage. The fat content and pH of the blended yoghurt decreased throughout the storage period, whereas the titratable acidity increased. The reduced fat content of the blended yoghurt is beneficial for extending its shelf life because the rate of rancidity is very low. Moreover, high titratable acid and the low pH of yoghurt are desirable because spoilage and pathogenic microorganisms struggle to grow in a highly acidic environment. In terms of sensory evaluation, yoghurt incorporated with 20% BGN had higher scores for taste and overall acceptability.

The use of bioprocessed BGN flour in food products is still lacking; however, Chinma et al. [198] added up to 30% of germinated BGN flour to wheat bread. The results showed that germinated BGN flour improved the protein, ash, fat, and dietary fibre content of wheat bread. Furthermore, the total phenolic and flavonoid contents, essential amino acids (except phenylaline), and protein digestibility of wheat bread were improved. The specific volume of the bread was not affected by the addition of 15% germinated BGN flour. The addition of up to 15% germinated BGN flour had no significant impact on the specific volume of wheat bread, whereas it decreased with 20% BGN flour inclusion. The sensory evaluation demonstrated that bread added with 20% germinated BGN flour had higher scores for taste, aroma, and overall acceptability. This study showed that bioprocessed BGN flour can be added at higher percentages than raw BGN flour to baked products.

## 7. Future Perspective and Conclusions

Bambara groundnut is rich in protein, carbohydrates, dietary fiber, and amino acids and contains a significant amount of minerals. Furthermore, BGN flour contains metabolites with potential health benefits, including polyphenols, flavonoids, sterols, and ketones. This demonstrates that BGN can be used as a functional ingredient in various food products. However, the hard-to-cook and hard-to-mill phenomenon, the presence of ANFs, and the beany flavor limit the utilization of BGN flour. Bioprocessing is the most common way to remove ANFs from BGN grains. Many traditional bioprocessing methods, such as soaking, dehulling, roasting, germination, cooking, malting, and fermentation, have been used to reduce ANFs in BGN flour. Bioprocessing, such as germination, malting, and fermentation, has been demonstrated to increase protein content and enhance technological characteristics and sensory evaluation by reducing the beany flavor, which is common in BGN. An increase in the polyphenol content and antioxidant activity is related to the germination and fermentation of legumes. Furthermore, novel bioprocessing techniques, such as ultrasonication, micronization, and infrared, have been utilized to improve the nutritional quality and functional properties of BGN flour. The consumption of whole BGN grains has prospective benefits for patients with diabetes mellitus, Alzheimer’s disease, cardiovascular diseases, arthritis, and others. This claim is supported by in vitro ACE activity, DPP-IV inhibitory activity, and antioxidant assay of BGN protein hydrolysates and peptides. In general, this review underscores the impact of bioprocessing on the nutritional composition, antinutrients, functional properties, and metabolism of BGN, thereby providing valuable information to optimize its consumption and usage in food products. Future research should focus on the impact of novel technologies such as high-pressure processing and ohmic heating on protein digestibility, antinutrients, amino acids, minerals, fatty acid profile, volatile compounds, and metabolites of BGN flour. This knowledge will encourage the broader use and application of bioprocessed BGN flours in different food products. Despite the promising results of processed BGN flour, some limitations should be acknowledged. The quality of processed BGN flour can vary because of variety differences, seasonal changes, and growing conditions, which may affect the consistency of the final product. Moreover, scale-up production may present challenges to maintaining the same quality characteristics of processed BGN flour highlighted in this study. Furthermore, the strong beany flavour of BGN might pose challenges when the flour is added to food products. Thus, its incorporation into food products should be at a lower level to warrant acceptability by consumers.

## Figures and Tables

**Table 2:** Influence of bioprocessing on the reduction level of ANFs of Bambara groundnut.

Processing Methods	Oxalate (mg/100 g)	Tannins (mg/100 g)	Phytate (mg/100 g)	Trypsin Inhibitors (mg/100)	Saponin (mg/100 g)	References
Roasting	Decreased (56.76 and 4.97)	Decreased (73.68)	Decreased (49.90, 30.59, and 1.98)	Decreased (73.42, and 37.51)	-	[33,49,50]
Soaking	Decreased (97.44)	Decreased (99.39)	Decreased (68.25)	Decreased (43)	Decreased (63.81)	[33,51]
Boiling	Decreased (81.30 and 23.34)	Decreased (73.68)	Decreased (5.91)	Decreased (67.31)	Decreased (36.67)	[14,51]
Fermentation	Decreased (59.11)	-	Decreased (25.58)	Decreased (75)	-	[52]
Germination	**-**	Decreased (63.74, 20.97, and 18.92)	Decreased (51.16, 24.71, and 15.91)	Decreased (73.75, 17.21, and 27.47)	-	[36,52,53]
Malting	Decreased (84.62)	Decreased (66.67)	Decreased (34.94)	Decreased (93.51)	Decreased (48.81)	[51]
Dehulling	Decreased (21.65 and 92.54)	Decreased (50)	Decreased (2.45 and 54.75%)	Decreased (5.32)	-	[18,39]

Values inside the brackets indicate the reduction level of ANFs of BGN as a percentage (%).

**Table 3:** Effect of bioprocessing on the functional properties of Bambara groundnut.

Processing Methods	WAC (%)	OAC (g/g)	FC (%)	FS (%)	EC (%)	BD (g/mL)	References
Roasting	Increased (81.04)	No change	Decreased (27.93)	-	Decreased (40.85)	Decreased (7.5)	[19,50,56]
Soaking	Increased (33.20)	Increased (2.09)	Increased (57.60)	-	Decreased (18.76)	Decreased (7.94)	[19,34,56]
Boiling	Increased (47.43)	-	Decreased (17.59)	-	Decreased (39.09)	Decreased (20)	[19]
Fermentation	Increased (16.18)	Decreased (13.61)	Decreased (45.19)	Increased (38.58)	Decreased (18.38)		[35]
Germination	Increased (42.5)	Decreased (18.67)	Increased (8.89)	Decreased (24.30)	Increased (2.96)	Decreased (40.26)	[36]

Values inside the brackets indicate functional properties of BGN as a percentage (%). WAC: Water absorption capacity; OAC: Oil absorption capacity; FC: Foaming capacity; FS: foaming stability; BD: Bulk density.

**Table 4:** Effect of processing methods on selected metabolites of Bambara groundnut.

Processing Method	Compounds	Detected or Destroyed	Increase or Decrease	Functions	References
Steeping	Polyphenols	-	- Increased	Possess anti-cancer, neuroprotective, antioxidant, and cardiovascular properties	[11,147]
Infrared	1. 9-Octadecene and Dodecanoic acid 2. Glycerol tricaprylate 3. 3-tert-Butyl-4-hydroxyanisole	1. Detected 2. Detected 3. Detected		Dodecanoic acid serves as a source of energy. Glycerol tricaprylate lowers the quantity of dangerous gut microbes and the amounts of inflammatory factors.	[11,148,149]
Fermentation	1. Esters 2. Dicyclohexyldisulfide, Benzene acetaldehyde, ß-Sitosterol, 2-Pyrrolidone carboxylic acid, 2-Hydroxybutyric acid, 4-Aminobutanoic acid, Azelaic acid, and 3-Pyridinol 3. Citric acid	- 2. Detected 3. ND	1. Increased	Dicyclohexyldisulfide is an industrial chemical that is frequently utilised to flavour foods. ß-Sitosterol is used to treat patients with malignancy or those with high cholesterol level.	[11,145,150,151]
Malting	1. Esters 2. Benzene acetaldehyde; (1H) Pyrrole-2-carboxaldehyde, 4-(trichloroacetyl)-; (Hexahydropyrrolizin-3-ylidene)-acetaldehyde 3. 3-tert-Butyl-4-hydroxyanisole 4. Glycerol 5. Bis(2-ethylhexyl) phthalate	- 2. Detected 3. Detected 4. ND 5. Detected	1. Decreased	Benzene and acetaldehyde inhibit microbial growth and are used to add aroma to food products. 3-tert-Butylhydroxyanisole (3-BHA) is a type of antioxidant that may modulate chemical oncogenesis. Bis(2-ethylhexyl) phthalate protects against harmful microbes that cause illnesses and regulates a variety of biological vectors.	[11,145,152,153,154]
Roasting	Esters, phenols, benzenes, and carboxylic acids		Decreased		[11]
Dehulling	1. Flavonoids (Catechin hexoside A, and B, Catechin, Quercetin-3-O-glucoside, Quercetin, Rutin, Medioresinol) 2. Phenolic acids (2,6-Dimethoxybenzoic acid, Protocatechuic acid, Vanillic acid, Syringic acid, Syringaldehyde, Gallic acid, Trans-cinnamic acid, p-coumaric acid, Caffeic acid, and Ferulic acid). 3. Syringic acid	- - 3. Destroyed	1. Decreased 2. Decreased -	Flavonoids possess positive well-being effects as they chelate trace elements that participate in the generation of free radicals, neutralising reactive oxygen scavengers or defending against antioxidants.	[49,155]
Ultrasonication	1. Butanal, 2. D-Glucuronic acid 3. Lactic acid, citric acid, phosphoric acid, and Glycerol monostearate	1. Detected	2. Decreased 3. Increased	Lactic acid improves food safety and nutritional content of products while also having a good effect on digestion and entire gastro-intestinal wellness.	[145,156,157]
Cooking	1. Catechin		1. Increased (Cream, orange and purple) 2. Decreased (Brown)	Citric acid inhibits viruses from replicating by raising the antioxidant levels as well as protecting intestinal wall by strengthening junctions that are tight and decreasing inflammation.	[14]
	2. Epicatechin		1. Increased (Cream, ad purple) 2. Decreased (Orange and brown)	Cardio and neuro protective, and anticancer	[14]
	3. Procyanidin		1 Increased (Brown and purple) 2. Decreased (orange and cream)	Anti-inflamatory and antidiabetic activity	[14]
	4. Citric acid		1. Increased (Cream, orange, and purple 2. Decreased (Brown)	Anti-inflammatory, anticancer, cardioprotective	[14]

Compound numbers in the “Detected or Destroyed” and “Increase or Decrease” columns correspond directly to those in the “Compound” column. For example, if compound 1 is listed, it will appear as 1 in the relevant column (s) to indicate its specific behavior (detected, destroyed, increased, or decreased) as affected by processing methods. ND—Not detected; - not determined.

## Data Availability

No new data were created or analyzed in this study. Therefore, data sharing does not apply to this article.
